# Exploration of the nutrition knowledge among general population: multi—national study in Arab countries

**DOI:** 10.1186/s12889-023-15791-9

**Published:** 2023-06-19

**Authors:** Hammam Bany-yasin, Abdellatif A. Elmor, Basant Khaled Ebrahim, Asaad Ayoub Mohamed Ahmed, Mohammad Roujan Alarachi, Lina Abedalqader, Rawan Amer, Ahmad Mohammad Samer Alyousef, Yasmine Fawaz Alhajeh, Abdullah Alyoussef, Hadeer Ahmed Mohamed Ahmed Eid, Maab Mohamed Elsayed, Eman D. El Desouky, Hosni Khairy Salem, Marwa Rashad Salem

**Affiliations:** 1 Kasralainy Faculty of Medicine, Cairo University, Cairo, Egypt; 2 Public Health Faculty of Medicine, Zagazig University State Country, Zagazig, Egypt; 3 Epidemiology and Biostatistics, National Cancer Institute, Cairo University, Cairo, Egypt; 4 Urology Faculty of Medicine, Cairo University, Cairo, Egypt; 5 Department of Public Health and Community Medicine, Faculty of Medicine, Cairo University, El Malek El Saleh, PO Box: 109, Cairo, 11559 Egypt

**Keywords:** Knowledge, Nutrition, Survey, Arab countries, Background

## Abstract

**Background:**

Knowing what to eat and realizing the significance of healthful eating habits are among the important steps to promoting eating behavior. The current study aims to assess the nutrition knowledge (NK) among a convenient sample in four different countries, determine the association between different demographic factors and NK, and investigate the need for future interventions on nutrition in the four selected countries.

**Methods:**

A cross-sectional multi-national survey study among a convenient sample of 8,191 subjects from Egypt, Syria, Saudi Arabia, and Jordan who undertook surveys between January 2019 and January 2020. A pre-tested interview questionnaire was utilized for data collection from study participants. It included three sections: i) Sociodemographic characteristics:. ii). Section two included twenty-one questions related to NK.. iii). Section three included one question about NK sources.

**Results:**

About three-quarters showed inadequate nutrition knowledge (73.1%). Youth (15–24 yrs.) were more dependent on social media, with 87% using it as a primary source of NK, while adults (≥ 25 yrs.) demonstrated that 43% of them used social media. In contrast, TV was more prominent among them, with participants’ characteristics such as living with parents, body mass index, and country of residence showing no association with NK. However, female sex, education, and reading nutrition articles are significantly correlated with adequate knowledge (*p* < 0.001). Significant predictors of satisfactory knowledge were age, sex, education, living with parents, and reading nutrition articles.

**Conclusion:**

The study revealed low levels of NK indicating an urgent need to implement educational programs to promote nutrition knowledge. As NK is a modifiable determinant of diet intake and can positively impact the need for developing strategies in counselling and raising awareness among the general population to improve their health status.

## Background

Nutrition is an important element that influences the quality of human life. Nutritional status is a substantial health indicator for assessing a country’s health standard and morbidity pattern [1]. Dietary habits and nutritional knowledge (NK) are very important for humans to have a healthy lifestyle especially for young adults [2]. Rapid changes and transitions in the life of a young adult is often characterized by poor nutrition and unhealthy weight gain [3].

Arab countries suffer from a high rate of obesity, as the prevalence of overweight and obesity among adolescents in Arab countries ranges from 18 to 44% [4]. Moreover, there was a recent report illustrating the dramatic increase of diabetes in most Arab countries and efforts should be made with the goal of overcoming this problem [5]. Risk factors for non-communicable diseases (NCDs) include; smoking, high blood pressure, overweight/obesity, high blood cholesterol, physical inactivity and inadequate nutrient intake [6]. Hence, promoting healthy lifestyles (adequate nutrition and physical activity) can prevent obesity and control chronic non-communicable diseases [7], which contribute to 60% of overall morbidity and mortality in most Arab countries [8].

Diet quality is influenced by NK, as nutritional knowledge is a prompting factor for the selection of a healthy diet [9]. Ruthsatz and Candeias [10] stated that NK may improve healthy food habits as well as consumption habits that may alleviate risk of developing NCDs. In another study, NK positively improved the use of food labels, a healthy food intake pattern, especially eating vegetables, and meat [11].

Assessment of NK is fundamental to determine what is needed to enhance inadequate food habit modifications and to promote nutritional status alterations [12]. NK is essential when an individual wants to improve their diet [10, 13]. In theory, information, motivation, and behavioral skill (IMB) are three concepts that interact with each other, resulting from a change in behavior. IMB has been widely used and proven to be a useful model in improving healthy practices [14].

Data on NK in the Arab world are limited since few studies have addressed this research problem. According to Musaiger et al. [15], 73.3% of Syrian and 71.5% of Jordanian students identified their lack of NK as significant barriers to healthy nutrition [15]. In Saudi Arabia, a study conducted by Al-Almaie [16] revealed that the dietary knowledge of both male and female students on the dangers of unhealthy foods and the benefits of fiber-rich diets is unsatisfactory.

Interpreting knowledge as well as knowledge impact on society is central to the development of dietary behavior change strategies [17–19]. To set up community-specific intervention programs, leading to the enhancement of lifetime excellent nutritional habits and thus good health, data on nutrition knowledge are required [17, 18].

Food and nutrition literacy is one of many factors contributing to the prevalence rates of malnutrition and health outcomes. A recent review emphasized that there is a current dearth of data, policies, and programs regarding food literacy and nutrition literacy in the Mediterranean Eastern region; a region plagued by high rates of malnutrition and nutrition-related disorders [19].

It is important to conduct this study in low-, middle- as well as high-income countries to identify the existing different levels of NK [19]. Appropriate health education interventions can increase awareness of simple public health nutrition messages in order to initiate positive behavior changes at a community-wide level [20, 21]. Plenty of evidence shows that adequate levels of food and nutrition literacy are positively associated with food selection, food preparation, eating habits, and diet quality [21].

The current study aimed to assess the NK among a convenient sample in four countries, to determine the association between different selected demographic factors and NK, and to explore the need for interventions.

### Research question

In the general population of Egypt, Syria, Saudi Arabia and Jordan,what is the level of nutrition knowledge?

## Methods

### Aim

To assess the NK among a convenient sample in four countries, namely Egypt, Syria, Saudi Arabia, and Jordan, to determine the association between different selected demographic factors and NK, and to explore the need for further interventions.

### Study setting and design

A cross-sectional, multi-national survey was conducted among a sample of the general population from four countries. The selection of participating study countries was based on identifying potential survey partners interested in the study. Overall, four countries showed their willingness to participate: Egypt, Syria, Saudi Arabia, and Jordan. In addition, according to the Global Nutrition Report, Egypt and Syria were burdened by a triple burden of malnutrition (overweight, anemia, and stunting), and Saudi Arabia and Jordan by a double burden of malnutrition (overweight and anemia).A country was considered ‘burdened’ by a malnutrition indicator depending on whether the national prevalence was greater than a certain cut-off. Stunting was measured in children under age 5, and its burden limit was 20% or more. Anaemia among women of reproductive age (15–49 years) had the same 20% or more cutoff, and for overweight women (18 +), this was 35% or more [22].

### Sample size and sampling technique

A convenient consecutive sample of 9,000 participants was reached and responded to on-street survey interview questionnaires. (Widely used in marketing research). In total 8267 agreed to participate (response rate 91.8%). Finally, 8191 (91.0%) forms were revised for completeness and logical consistency. The number of participants from each country was 2,483 (Egypt), 2,110 (Syria), 1,895 (Saudi Arabia), and 1,703 (Jordan). Data were collected between January 2019 and January 2020.

Participants aged > 18, apparently healthy (By asking participants about having any chronic conditions such as DM, HTN, CVD, which were considered as unhealthy participants in this study). Those who responded to the screening question at the start of the interview by yes about if they have a chronic disease, or were on diet, or whether they were health care providers, were excluded.

Participants were recruited in the four countries in crowded locations such as malls, around clubs. Particular streets were chosen based on the site of the high traffic enumeration via tossing in. Subjects were approached and informed of the study objectives.

### Study questionnaire

A pre-tested street survey interview questionnaire included three sections:

i) Section one included background characteristics: age in years, gender, education, country of residence, and living with parents or not.
ii) Section two included 20 questions related to NK. NK was evaluated based on an adapted consumer NK scale version (CoNKS) by Dickson-Spillmann et al. [23]. The scale uses procedural as well as declarative nutritional knowledge questions to assess NK. The final item set entailed questions about declarative NK on nutrient calorie as well as contents (For instance, ‘‘The same amount of sugar and fat contains equally many calories’’) in addition to comparisons of food (For example, ‘‘A salad dressing made with mayonnaise is as healthy as the same dressing made with mustard’’); besides questions about procedural NK on the related contribution of various food groups to healthful nutrition (For example, ‘‘For healthy nutrition, dairy products should be consumed in the same amounts as fruit and vegetables’’), on the fat role (like ‘‘Fat is always bad for your health; you should therefore avoid it as much as possible’’), and finally on fruit benefit as well as consumption of vegetables (For instance, ‘‘To eat healthily, you should eat less fat. Whether you also eat more fruit and vegetables does not matter’’). The knowledge tool consists of three subscales: one scale for procedural NK (Seven items, thus Zero–Seven points), one scale for nutrient content declarative knowledge (Seven items, Zero-Seven points), and one scale for calorie content declarative knowledge (Six items, Zero-Six points) A 20 closed-ended format were employed with ‘Yes’, ‘No’, and ‘don’t know’ options. The correct response received a score of one, while the incorrect response received zero. The total score ranged from 0 to 20.
iii) Section three had one question about the sources of NK and included multiple option formats that asked about educational courses, health care providers, the university, mass media, family, social media, and relatives.

### Anthropometric measurements

Self-reported weights and heights of the participants were utilized to estimate body mass index (BMI). We classified subjects as healthy weight (BMI 18.5 to 24.9 kg/m2), overweight (BMI 25.0- to 29.9), obese (BMI > 30-), as well as underweight (BMI < 18.5) [24].

Data collection was performed by trained researchers after receiving standardized training on how to conduct the survey.

### Pilot testing

In larger populations, surveys, CoNKS can be considered a quick and efficient tool for measuring NK [23]. We tested the questionnaire on 100 participants in the four countries to evaluate comprehension as well as clarity of questions. As a result, it was deemed appropriate for use in the current study. Nonetheless, certain scale changes were deemed essential. This entailed food terminology usage that is more widespread in the four nations (For example, cheddar cheese instead of Emmental and Swiss cheese).

### Final questionnaire

The questionnaire consisted of 20 items with an estimated reliability coefficient of (Cronbach’s alpha = 0.797), in addition to inquiry about sources of knowledge that was evolved during the pilot. The researchers established face and content validity before conducting the study by reviewing the questionnaire items with faculty staff members. Three questions about the recommended dietary allowance of macronutrients were deleted due to non-specific responses.

English was the original language of the questionnaire items, and it was then translated into Arabic by two experts followed by a translation into English by other experts.

### Statistical analysis

The pre-coded data were fed into the computer via the Statistical Package of Social Sciences (SPSS) version 24.0 (SPSS Inc. IBM, U.S.A.) to be statistically analyzed. We presented data as frequencies and percentages for categorical data; the Chi-square test was utilized for comparison when appropriate. For quantitative variables, mean, median, standard deviation, as well as interquartile range, were used for expression; an independent sample t-test was utilized for comparison. Total knowledge score was computed (a total of 21 questions), correct responses were assigned a score of 1, whereas incorrect or not sure responses were assigned zero.

NK scores can range from 0–20 points. The total knowledge of respondents was classified based on the modified Bloom’s cut-off point as follows: good for scores of 80–100% (16–20 points), moderate for scores of 50-79% (10–15 points), poor score of< 50% (< 10 points) then recategorized into satisfactory and unsatisfactory (<16 points) [25].

The researchers pooled the scores of all questions in each section in each respondent and then divided them by the overall participant number. All scores were then multiplied by 100 and presented as percentages.

A forward stepwise logistic regression model was performed to explore predictors of satisfactory knowledge, all background characteristics variables (age, sex, country, education, living with parents, BMI, and reading nutrition articles) were entered in the first step.

We considered all statistical tests to be statistically significant at *P* < 0.05.

## Results

The study included 8,191 participants who were residents in Egypt (30.3%), Jordan (25.8%), Saudi Arabia (23.1%), and Syria (20.8%). Their mean age was 21.6 ± 3.9 years, with a range of 18 to 59 years. Females represented about two-thirds (65.2) % of the sample. Most of the interviewed participants (95.6%) were educated. The prevalence of overweight and obesity were 24.7% and 12.2%, respectively, as reported by the study participants. About three-quarters showed inadequate NK (73.1%) as illustrated in Table 1.

**Table 1:** Baseline characteristics of the enrolled participants

Background Characteristics	(*n* = 8191)
**Age** *(Mean* ± *SD)*	21.6 ± 3.9
**Sex** *n,( %)*	
- Male	2853(34.8)
- Female	5338(65.2)
**Country** *n,( %)*	
- Egypt	2483 (30.3)
- Jordan	2110 (25.8)
- Saudi Arabia	1895(23.1)
- Syria	1703 (20.8)
**Education** *n,( %)*^b^	
- Educated	7832 (95.6)
- Uneducated	359 ( 4.4)
**Living with parents n*,( %)***	
- Yes	6908 (84.3)
- No	1283 (15.7)
**Body mass index (BMI)** *n,( %)*	
- Underweight	626(7.6)
- Normal	4549 (55.5)
- Overweight	2024 (24.7)
- Obese	992(12.2)
**Knowledge level** *(n, %)*	
- Satisfactory	2204 (26.9)
- Unsatisfactory	5987(73.1)
**Weight (Kg) (***Mean* ± *SD)*	68.3 ± 17.7
**Height ( cm)** *(Mean* ± *SD)*	166.6 ± 9.3
**Body mass index** *(Mean* ± *SD)*^a^	24.5 ± 5.4
**Knowledge score** *Median (IQR)*	14 (11–17)

^a^BMI was calculated based on participant’s self-reported weight and height

^b^Education means has formal education level ( Primary – preparatory – high school, university degree and post graduate) In the current study our study participants were high school and university graduates

**Fig. 1:**
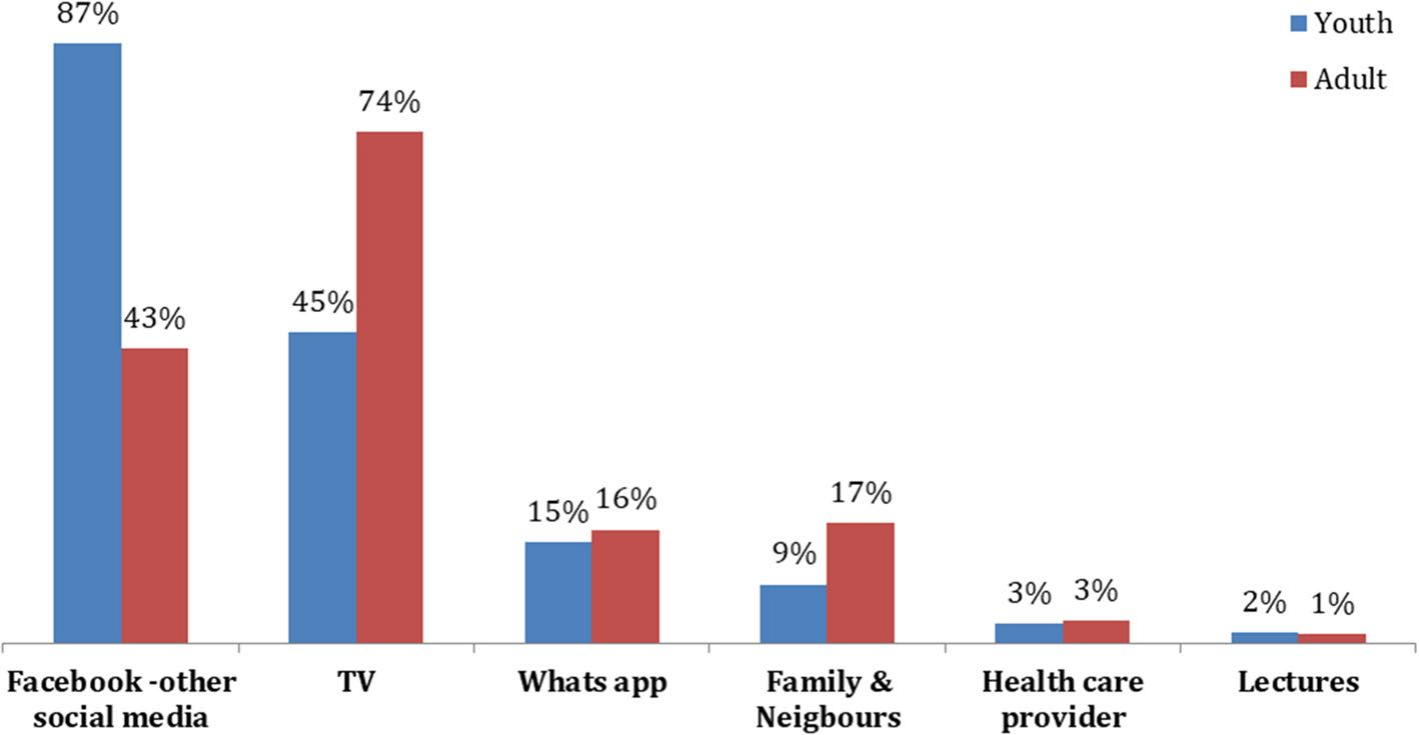
Percent distribution of study participants by age group and source of nutrition knowledge. Youth (15-24 yrs.), while adults (≥ 25 yrs.)

Youth (15–24 yrs.) were more dependent on social media: 87% of them used it as a primary source of NK, while 43% of adults (≥ 25 yrs.) used social media. In contrast, TV was more prominent among them (Fig. 1).

As displayed in Table 2, NK was similar between different countries. Age was statistically significant, but the difference is small and negligible, and female participants were statistically higher in Jordan rather than in the other countries. BMI was higher in Saudi Arabia than in the other countries, whereas educated participants were higher Knowledge level (Kl) were comparable between different countries. Were comparable between different countries. Although there is little statistical significance regarding some demographic characteristics, this may not be clinically significant because of the large sample size.

**Table 2:** Comparison between the four countries regarding baseline characteristics and Knowledge level (*n* = 8191)

		**Egypt** ***n* = 2483**	**Saudi Arabia** ***n* = 1895**	**Syria** ***n* = 1703**	**Jordan** ***n* = 2110**	***p* value**
**Age (yrs.)**^**a**^	Mean ± SD	22.0 ± 3.6	22.4 ± 5.2	21.2 ± 3.5	20.7 ± 3.2	** < 0.001**
**Sex**^**a**^	Male	897(36.1)	801(42.3)	642(37.7)	513(24.3)	** < 0.001**
	Female	1586(63.9)	1094(57.7)	1061(62.3)	1597(75.7)	
**BMI**^**a**^	Mean ± SD	24.6 ± 5.1	26.1 ± 6	23.2 ± 4.7	23.9 ± 5.4	** < 0.001**
**Education**^**a**^	Un Educated	73(2.9)	106(5.6)	93(5.5)	87(4.1)	** < 0.001**
	Educated	2410(97.1)	1789(94.4)	1610(94.5)	2023(95.9)	
**living with parents**	Yes	1970(79.3)	1599(84.4)	1462(85.8)	1877(89.0)	** < 0.001**
	No	513(20.7)	296(15.6)	241(14.2)	233(11.0)	
**Reading nutrition article**	Yes	1424(57.3)	1126(59.4)	965(56.7)	1258(59.6)	0.152
	No	1059(42.7)	769(40.6)	738(43.3)	852(40.4)	
**knowledge Level**	Unsatisfactory	1795(72.3)	1380(72.8)	1281(75.2)	1531(72.6)	0.164
	Satisfactory	688(27.7)	515(27.2)	422(24.8)	579(27.4)	

^a^All groups are statistically significant from each other’s

Participants’ features, such as living with parents, body mass index, and country of residence, showed no association with nutrition-related knowledge. However, female gender, education, and reading nutrition articles were significantly associated with adequate knowledge (*p* < 0.001) as illustrated in Table 3.

**Table 3:** Relation between different background characteristics and nutrition related knowledge (*n* = 8191)

Background Characteristics	Unsatisfactory knowledge level *n* = 5987	Satisfactory Knowledge level *n* = 2204	*P* value
**Age (yrs.)** *(Mean* ± *SD)*	21.3 ± 3.8	22.1 + 4.4	* < 0.001
**Body mass index** *(Mean* ± *SD)*	24.5 ± 5.5	24.4 ± 5.2	0.454
**Sex (***n, %)*			
- Male	2326 (81.5)	527 (18.5)	* < 0.001
- Female	3661 (68.6)	1677 (31.4)	
**Country** *(n, %)*			
- Egypt	1795 (72.3)	688 (27.7)	0.164
- Saudi Arabia	1380 (72.8)	515 (27.2)	
- Syria	1281, (75.2)	422 (24.8)	
- Jordan	1531, (72.6)	579 (27.4)	
**Education** *(n, %)*			
- Educated	5678 (72.5)	2154 (27.5)	* < 0.001
- Un educated	309 (86.1)	50 (13.9)	
**Living with parents *(n, %)***			
- Yes	5035 (72.9)	1873 (27.1)	0.330
- No	952 (74.2)	331 (25.8)	
**Reading nutrition articles** *(n, %)*			
- Yes	3233 (67.7)	1540 (32.3)	** < 0.001**
- No	2754 (80.6)	664 (19.4)	

^*^Statistical significance level *P* < 0.05

^a^Education means has formal education level ( Primary – preparatory – high school, university degree and post graduate) In the current study our study participants were high school and university graduates

By multivariable logistic regression, the significant predictors of satisfactory knowledge were age, sex, education, living with parents, and reading nutrition articles as displayed in Table 3.

Age was directly correlated with the level of NK (with increasing age, knowledge increased); females had more knowledge than males with OR 1.97 with 95% CI (1.76–2.22). Educated participants were 2.5 times more knowable than uneducated. Participants living with parents and who read nutrition articles had 1.18 and 1.81 more knowledge than those who do not live with parents or do not read articles, respectively, as shown in Table 4

**Table 4:** Logistic Regression Model for Predictors of knowledge among the enrolled participants

	**B(S.E.)**	***p* value**	***OR(95%CI)***
Age	0.05(0.01)	< 0.001	1.05(1.03–1.06)
Sex	0.68(0.06)	< 0.001	1.97(1.76–2.22)
Education	0.9(0.16)	< 0.001	2.46(1.8–3.35)
living with parents	0.16(0.07)	0.028	1.18(1.02–1.35)
Reading nutrition article	0.6(0.05)	< 0.001	1.81(1.63–2.02)
Constant	-5.12(0.37)	< 0.001	0.01

*B* Regression coefficients, *SE* Standard error of the coefficient, *OR* Odds Ratio, 95% CI for OR = 95% confidence interval for the Odds Ratio. *P*<0.05 is considered significant

## Discussion

The current study demonstrates that the majority of the study participants had inadequate nutrition knowledge (73.1%). This finding is consistent with Musaiger et al. [15], who reported that 73.3% of Syrians, 72.1% of Egyptians, and 71.5% of Jordanian students identified a lack of NK as a significant barrier to healthy nutrition. Unsatisfactory knowledge might be illustrated by the fact that nutrition as a science is newly introduced, with no formal nutrition education instruction being introduced in schools. At the university level, it is studied only at specialised institutions. The current study results are lower than those indicated by a study of young adults in the Accra metropolis, where more than half of the subjects had elevated scores on the assessment of basic NK [11]. The difference between the findings from the two studies can be explained by the fact that the majority of participants in the that study were students (66.1%) and had completed their senior high school level education. We observed that significant predictors of satisfactory knowledge were age, sex, being educated, living with parents, and reading nutrition articles.

The results indicate that the main source of knowledge is social media, followed by mass media. This is expected as younger people are less likely to cling to tradition and are more affected by social media. Consequently, professionally designed nutrition education programmes have to be introduced early in life through school, and subsequently via the media in order to counteract faulty nutrition-related knowledge. Hendrie et al. [26] observed a detectable relationship between NK and age. However, Dickson-Spillmann et al. [13] detected a negative relationship. Parmenter et al. [27] reported a curvilinear relationship, where middle-aged groups had elevated knowledge in contrast to younger and older subjects. It is noteworthy that the studies differed significantly in how age groups were defined, which limits comparability.

In the current study, the female gender was significantly associated with adequate NK. This could be attributed to the fact that females tend to be more aware of their appearance than males [28]. The finding is consistent with what was demonstrated by a similar study by Koch et al. [19], as greater NK was observed in females, and this finding supports the assumption that females are more interested in aesthetic aspects, leading them to search for NK. In a study conducted by Labban et al. [29] among university students in Syria, females scored higher for nutrition knowledge than males did [30].

Our study showed an association between NK and educational level, which is compatible with a previous study performed by Koch et al. [19], where NK was more elevated in the higher education groups. This clearly demonstrates that education is a critical tool for acquiring NK. This finding underscores the importance of conducting nutrition awareness campaigns in schools and universities. In addition, a study conducted by Labban et al. [27] among Syrian university students revealed that, when compared to students who were enrolled in non-health-related programmes, those who took health-related courses performed better.In the current study, an elevated pervasiveness of obesity, as well as being overweight, was observed. Inadequate NK may contribute to increasing the pervasiveness of obesity and overweight among the participants, but this relationship is not statistically significant. A review by Spronk et al. [31] illustrated that multiple studies have detected substantial associations between high NK and healthier food consumption, particularly elevated consumption of vegetables and fruit. A study by Musaiger et al. [15] to investigate the prevalence of obesity as well as overweight among adolescents in seven Arab countries utilising an identical reference standard demonstrated that overweight among males was higher among Kuwaiti adolescents (25.6%) than Jordanians (21.6%), and finally Syrian (19.7%) adolescents. Concerning females, the highest prevalence of overweight among adolescents was reported in Libya (26.6%), then Kuwait (20.8%), and finally Syria (19.7%) Concerning obesity, Kuwaiti adolescents demonstrated the highest pervasiveness of obesity for both males (34.8%) and females (20.6%).

In the current study, social media, such as Facebook, was the most common information source regarding nutrition among youth, while healthcare providers were reported by the minority. This finding is compatible with that reported by a study performed on young adults in the Accra Metropolis [17] in which healthcare professionals were regarded as the most reliable source of nutrition information. However, a very limited number of respondents consulted them. The Internet, acquaintances, family, television, as well as books, have all been highlighted as common sources of NK in the literature [32, 33]. As a result, additional information on health information acquisition behaviours is required, especially in economically developing nations in which internet use is rapidly becoming a prevalent tool for acquiring information. Hence, social media interventions can be successful as they increase awareness of public issues, encourage civilian involvement, enhance health systems, become a collaborative space for science broadcasting, support health policies, and encourage healthy behaviors [34]. It has many advantages, such as helping people to increase their physical activity levels [35], reducing their sugar and fat consumption, and delivering nutritional or diabetes education [36].

However, misleading information is a major challenge in using social media [37], and it is usually supported by fake non-scientific accounts [38]. Consequently, academia and trustful institutions should respond positively to the adoption of reliable social media accounts. Another challenge is the marketing finances of health institutions versus commercial companies; companies allocate huge funds for marketing on social media [39].

Traditional media (e.g., television) were the second most utilised information source in the current study. The information disseminated by traditional media is usually prepared for heterogeneous audiences and is not tailored to fulfil specific individual needs, in contrast to online resources that host like-minded individual communities to share health information of interest to a particular audience [29]. In a Saudi study conducted by Al-Almaie [16] addressing knowledge and sources of knowledge about health and disease, the following was revealed by both male and female students: television came in first place (58% and 61%), and then magazines (31% and 39%). Primary health care centre (PHCC) staff were the least commonly used sources of knowledge (17% and 16%).

## Conclusion

Unsatisfactory nutrition knowledge was evident among most participants. This finding emphasises the importance of engaging in nutrition awareness campaigns, and this could be done through social media, the most familiar source of nutrition knowledge among the study participants.

### Recommendations

We suggest future research and population-based surveys as well as NK programmes for further understanding of their NK levels and determinants. As NK is a modifiable determinant of diet intake and can positively impact the need for developing strategies in counselling and raising awareness among the general population to improve their health status,

### Limitations

The current study findings should be viewed with respect to the following limitations: The descriptive nature of the study and the use of non-probability sampling techniques However, the researchers conducted the current study to explore the situation in this new area of inquiry and to generate hypotheses, as little information is available regarding nutritional knowledge and literacy in the selected countries. It was not used to infer causal relationships. Nevertheless, the study provides more information on a poorly researched area and contributes to guiding future nutrition education programmes. The researchers were not aiming at generalising the study findings but just to explore the situation in the enrolled countries. However, the primary outcome of the current study was to assess nutrition knowledge among a sample of the general population. And even the actual measurements are not accurate, and we need to follow strict instructions to allow an accurate measurement of anthropometric measurements.

In large surveys, the measurement of weight and height is often not feasible due to financial, logistical, and human resource limitations, and self-reported values are used as an alternative. Easy to collect, self-reported values are prone to misreporting [40, 41]. According to a study conducted by Chia et al., 2023While self-reported body weight showed weaker agreement with actual measurements, particularly for obese and overweight individuals, BMI values derived from self-reported weight and height were accurate for 88.53% of the participants.

## Data Availability

The datasets used and/or analyzed during this study are available from the corresponding author upon reasonable request.
